# From bones to blood pressure, developing novel biologic approaches targeting the osteoprotegein pathway for pulmonary vascular disease

**DOI:** 10.1016/j.pharmthera.2016.06.017

**Published:** 2017-01

**Authors:** Sarah Dawson, Allan Lawrie

**Affiliations:** Department of Infection, Immunity and Cardiovascular Disease, Faculty of Medicine Dentistry and Health, University of Sheffield Medical School, Beech Hill Road, Sheffield S10 2RX,UK

**Keywords:** 5HT, 5 hydroxytriptamine, Apo2L, Apoprotein 2 ligand, ApoE, Apolipoprotein E, BMPR2, Bone morphogenetic protein receptor type 2, CAD, Coronary artery disease, CKD, Chronic kidney disease, DR4/5, Death receptor 4/5, DcR1/2, Decoy receptor 1/2, FGF-2, Fibroblast growth factor 2, HuDEMC, Human dermal microvascular endothelial cells, HMVEC, Human microvascular endothelial cells, HUVEC, Human umbilical cord vein endothelial cells, IL-1, Interleukin 1, kDa, Kilodalton, LDLR, Low-density lipoprotein receptor, OCIF, Osteoclastogenesis inhibitory factor, OPG, Osteoprotegerin, PAD, Peripheral artery disease, PAH, Pulmonary arterial hypertension, PASMC, Pulmonary arterial hypertension, RANK, Receptor activator of nuclear factor κ B, RANKL, Receptor activator of nuclear factor κ B ligand, TNF, Tumor necrosis factor, TRAF6, Tumor necrosis factor receptor associated factor 6, Pulmonary hypertension, Osteoprotegerin, Bone, Vascular, Biologics, Therapeutics

## Abstract

Osteoprotegerin (*tnfsf11b*, OPG) is a soluble member of the TNF superfamily originally described as an important regulator of osteoclastogenesis almost 20 years ago. OPG is a heparin-binding secreted glycoprotein that exists as a 55–62 kDa monomer or a 110–120 kDa disulphide-linked homodimer. Acting as a soluble decoy receptor for RANKL, OPG actively regulates RANK signalling, and thereby osteoclastogenesis. OPG has subsequently been shown to also be a decoy receptor TNF related apoptosis inducing-ligand (*tnfsf10*, TRAIL, Apo2L). TRAIL is a type II transmembrane protein that is widely expressed in a variety of human tissues, including the spleen, lung, and prostate. Through binding to TRAIL, OPG can inhibit TRAIL-induced apoptosis of cancer cells. More recently, OPG has been demonstrated to be secreted by, and influence, vascular smooth muscle cells phenotype particularly related to vascular calcification and pulmonary vascular remodelling. In pulmonary artery smooth muscle cell (PASMC) suppression of BMP, induction of 5-HT and IL-1 signalling have been shown to stimulate the release of OPG *in vitro*, which causes cell migration and proliferation. Patients with idiopathic PAH (IPAH) demonstrate increased circulating and tissue levels of OPG, and circulating serum levels predict survival. In pre-clinical models, OPG levels correlate with disease severity. Since OPG is a naturally circulating protein, we are investigating the potential of novel biologic antibody therapies to rescue PAH phenotype in disease models. Further pre-clinical and mechanistic data are forthcoming, but we believe current published data identify OPG as an exciting and novel therapeutic target in PAH.

## 1 Introduction

Since its discovery nearly 20 years ago, there have been significant advances in our understanding of the role of osteoprotegerin in health and disease. Osteoprotegerin, meaning “to protect bone,” was originally purified from human fibroblast conditioned media and described as the osteoclastogenesis inhibitory factor (OCIF) by Tsuda et al. (1997) because of its ability to inhibit bone reabsorption (Tsuda et al., 1997). Within the same year, Simonet et al. identified OPG as an important regulator of bone density after transgenic mice overexpressing OPG developed osteopetrosis and a decrease in osteoclast number (Simonet et al., 1997). Analysis of a foetal rat intestinal library revealed a 401-amino-acid-long, secreted cytokine with an N-terminus analogous to TNF receptor superfamily members (Simonet et al., 1997). We now know that OPG is a heparin-binding secreted glycoprotein belonging to the TNF receptor superfamily that exists as either a 55–62 kDa monomer or a 110–120 kDa disulphide-linked homodimer (Simonet et al., 1997, Zauli et al., 2007).

OPG contains a 21-amino-acid long signal peptide that is cleaved to generate the mature, 380-amino-acid-long form (Zauli et al., 2007). The OPG protein consists of seven structural domains; the function of all but one of these domains has been determined. Domains 1–4 are cysteine rich and share structural similarities with the TNF receptor extracellular domains, and are sufficient to abolish osteoclastogenesis. Domains 5 and 6 contain death domains, which share similarities with both the Fas and TRAIL death receptors. Domain 7 consists of 50 amino acids and contains the cys-400 residue that is essential for disulphide bond formation and dimerisation of OPG (Yamaguchi et al., 1998). Domain 7 may also play an important role in regulating the release and activity of OPG (Zauli et al., 2007).

## 2 The osteoprotegerin/receptor activator of nuclear factor κ B ligand/receptor activator of nuclear factor κ B axis in bone biology

**Fig. 1:**
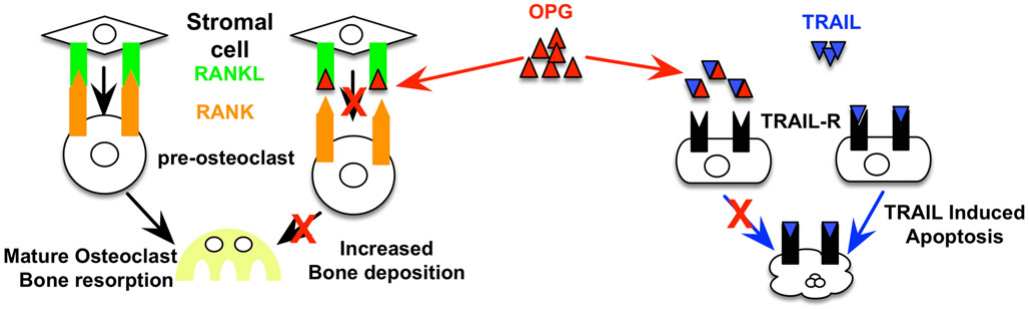
*Model of OPG regulation of bone remodelling and TRAIL-induced apoptosis.* OPG binds to RANKL expressed by stromal cells to prevent RANK–RANKL binding on pre-osteoclasts to regulate osteoclastogenesis. OPG can also bind to TRAIL and inhibit TRAIL binding to TRAIL receptors expressed on tumour cells. In doing so, OPG can protect against TRAIL-induced apoptosis.

The osteoprotegerin (OPG), receptor activator of nuclear factor κ B ligand (RANKL), receptor activator of nuclear factor κ B (RANK) axis plays an important role in bone remodelling and is critical for regulating bone density. OPG acts as a decoy receptor for RANKL to inhibit osteoclastogensis (Yasuda et al., 1998, Vitovski et al., 2007). Bone is continuously renewed through reabsorption at the trabeculae by osteoclasts, and new bone deposition by osteoblasts (Hofbauer and Schoppet, 2004, Boyce and Xing, 2007). Osteoclastogenesis requires binding of RANKL, a type 2 homotrimeric transmembrane protein expressed on mature osteoblasts, to its receptor, RANK, a type 1 homotrimeric transmembrane protein expressed on osteoclast precursor cells (Hofbauer and Schoppet, 2004, Boyce and Xing, 2007, Vitovski et al., 2007) (Fig. 1). Upon formation, this receptor ligand complex induces osteoclast formation, activation, and survival via NF-kB through recruitment of the adaptor protein, tumor necrosis factor receptor associated factor 6 (TRAF6) (Boyce & Xing, 2007) to prevent precursor differentiation into macrophages (Hofbauer and Schoppet, 2004, Boyce and Xing, 2007, Vitovski et al., 2007). NF-kB then translocates to the nucleus to induce c-Fos expression, which subsequently results in osteoclastogenic gene transcription. OPG, secreted from osteoblasts, acts as a decoy receptor for RANKL, preventing the RANKL–RANK binding, osteoclast activation, and subsequent bone reabsorption (Hofbauer and Schoppet, 2004, Vitovski et al., 2007). Post-natal OPG is critical for the maintenance of bone density and disrupted OPG expression *in vivo* results in the development of bone disorders (Bucay et al., 1998). OPG knockout mice exhibit osteoporosis due to excessive bone reabsorption (Bucay et al., 1998), and conversely, elevated OPG levels or inactive RANKL result in osteopetrosis due to reduced bone reabsorption (Simonet et al., 1997, Vitovski et al., 2007). In order to protect against excessive bone reabsorption, OPG mRNA is up-regulated during the normal process of bone formation (Tanaka et al., 2011). The critical role for the regulation of OPG has been highlighted by genetic studies showing that mutations in OPG, that affect expression levels, have been associated with juvenile Paget's disease (Whyte et al., 2002).

## 3 Osteoprotegerin and tumor necrosis factor (TNF)-related apoptosis-inducing ligand in tumour cell biology

As well as its role in bone biology, OPG also plays an important role in tumour cell biology as a decoy receptor for TRAIL (Emery et al., 1998). Tumor necrosis factor (TNF)-related apoptosis-inducing ligand (TRAIL, Apo2L) is a type II transmembrane protein that is widely expressed in a variety of human tissues, including the spleen, lung, and prostate. In humans, TRAIL has four transmembrane receptors: death receptor 4 (DR4, TRAIL-R1), DR5 (TRAIL-R2), decoy receptor 1 (DcR1, TRAIL-R3), DcR2 (TRAIL-R4), and the fifth, OPG. By binding to TRAIL, OPG has been shown to inhibit TRAIL-induced apoptosis of Jurkat cells, and TRAIL also represses OPG inhibition of osteoclastogenesis (Emery et al., 1998). Through interaction with TRAIL, OPG was also found to inhibit TRAIL-induced apoptosis of ovarian cancer cells (Cross et al., 2006), a process that occurs in an α_v_β_3_ integrin and α_v_β_5_ integrin-dependent manner (Lane et al., 2012, Lane et al., 2013). OPG has also been reported to prevent TRAIL-induced apoptosis of human microvascular endothelial cells (HMVECs), a process also requiring α_v_β_3_ (Pritzker et al., 2004) (Fig. 1).

Along with cancer cell survival, OPG has also been implicated in angiogenesis, a process required for the maintenance, development, and progression of tumours (Cross et al., 2006). OPG expression was identified in the endothelium of malignant colorectal, breast, and metastatic cancer tumours, but not in the endothelium of benign tumours or normal tissue. OPG induces human dermal microvascular endothelial cells (HuDMECs) to form cord-like capillary structure (Cross et al., 2006) and induces vessel-formation *in vivo* via heparin binding (McGonigle et al., 2008). More recently, work undertaken by Benslimane–Ahmim and colleagues has shown that OPG induces the migration and differentiation of endothelial colony-forming cells into cord-like structures, promotes fibroblast growth factor-2 (FGF2)-induced neo-angiogenesis *in vivo*, and increases endothelial colony-forming cell adhesion to fibronectin *in vitro* (Benslimane-Ahmim et al., 2013).

## 4 Osteoprotegerin and atherosclerosis and calcification

OPG has been well described for its critical role bone biology but also more recently in vascular biology. OPG is known to be widely expressed in a variety of tissues, including the human heart, kidney, placenta, and lung (Simonet et al., 1997). A variety of cells express and secrete OPG, including bone marrow stromal cells and cells belonging to the osteoblastic cell lineage, B cells, megakaryocytes, platelets, vascular endothelial cells, and vascular smooth muscle cells (Collin-Osdoby et al., 2001, Collin-Osdoby, 2004, Olesen et al., 2005, Li et al., 2007, Zauli et al., 2009, Condliffe et al., 2012). OPG has subsequently been implicated in a variety of processes and diseases, including atherosclerosis, vascular calcification, angiogenesis, and hypertension.

The development of calcified arteries alongside osteoporosis in OPG^−/−^ mice first revealed a role for OPG in vascular biology, and accumulating evidence supports a protective role for OPG against calcification (Bucay et al., 1998). OPG was also shown to have protective role in the progression and calcification of advanced atherosclerotic lesions in the innominate arteries of Apolipoprotein E (ApoE)^−/−^ mice, in so much that aged mice double deficient for ApoE and OPG developed larger and more complex atherosclerotic lesions (Bennett et al., 2006). Corollary, OPG treatment reduced vascular smooth muscle cell calcification *in vitro*, and subsequently, restoration of OPG reverses experimental calcification in pre-clinical models (Schoppet et al., 2011). High concentrations of calcium which induce calcification of vascular smooth muscle cells have also found to induce OPG mRNA expression in healthy vascular smooth muscles cells (Schoppet et al., 2011). There is, however, some controversy surrounding the role of OPG in vascular calcification. For example, concentrations of OPG equivalent to those measured in coronary artery disease (CAD) and chronic kidney disease (CKD) patient serum appear to have no measurable effect against calcification (Schoppet et al., 2011). Olesen et al. (2012) reported that OPG had no effect on vascular smooth muscle cells calcification. The role of OPG in vascular calcification in disease still remains elusive.

## 5 Osteoprotegerin and vascular cell phenotype

There is now accumulating evidence that OPG plays an even wider role in vascular biology. OPG induces the proliferation of vascular endothelial cells from a variety of vascular beds including HuDMEC and HUVEC (Cross et al., 2006), mediated through α_v_β_3_ and α_v_β_5_ integrins (Kobayashi-Sakamoto et al., 2008). OPG-induced HuDMECs and HUVECs proliferation has been shown to be mediated via a TRAIL-independent signalling mechanism (Cross et al., 2006). However, there are contradictions in the literature regarding the sensitivity of HuDMECs and HUVECs to TRAIL and there have been reports in the literature that show TRAIL can alter HUVEC and HuDMEC phenotype (Pritzker et al., 2004, Secchiero et al., 2004). In VSMCs, OPG has also been shown to induce the survival of serum-deprived aortic SMCs (Bennett et al., 2006), and the proliferation and migration of pulmonary artery SMC (Lawrie et al., 2008). Intriguingly, a microarray comparison study of early and late passage VSMCs identified OPG to be one of the most differentially expressed genes in senescence (Burton et al., 2009), providing evidence linking OPG expression with vascular cell phenotype.

OPG also binds to syndecan-1 (SDC-1) (Mosheimer et al., 2005, Baud'huin et al., 2013), and the OPG–SDC-1 interaction has been found to be important in monocyte migration (Mosheimer et al., 2004) and may therefore influence vascular inflammation.

## 6 Osteoprotegerin in cardiovascular disease

OPG has been implicated as a potential mediator and biomarker in a variety of cardiovascular diseases, particularly cardiometabolic conditions (Pérez de Ciriza et al., 2015) and pulmonary hypertension (Lawrie et al., 2008, Condliffe et al., 2012). In a recent patient study, hypertensive patients were shown to have elevated OPG levels compared to control patients (Stępień et al., 2011). Elevated OPG levels have also been observed within pericardium of malignant and non-malignant pericardial effusion (Karatolios et al., 2012), and there is also a positive correlation between the severity of peripheral artery disease (PAD) and plasma values of OPG (Ziegler et al., 2005). Expression of OPG and its ligand, RANKL, have been observed within the failing myocardium, and OPG, RANKL, and RANK are all up-regulated in experimental and clinical heart failure. Patients with severe aortic stenosis exhibit elevated systemic OPG expression and OPG, RANKL, and RANK mRNA expression is increased within ischemic areas of the left ventricle (Ueland et al., 2012). OPG plasma levels are also a predictor of asymptomatic coronary artery disease in type-2 diabetic patients and OPG plasma levels are also significantly higher in patients with systemic hypertension, decreased kidney function, and type 1 diabetic patients with nephropathy and signs of cardiovascular disease (Ueland et al., 2004, Avignon et al., 2005, Ueland et al., 2005, Rasmussen et al., 2006).

Although increased expression of OPG has been associated with cardiovascular disease, OPG is actually thought to play an important atheroprotective role as described above. Interestingly, pre-clinical bone marrow transplantation experiments from mice suggest both vessel wall-derived and bone marrow-derived OPG as sufficient to reduce both atherosclerotic lesion size and calcification in the innominate artery (Callegari et al., 2013). However, as with the literature describing the role OPG plays in calcification, there are conflicting reports regarding the role of OPG in atherosclerosis. High-fat diet fed LDLR^−/−^ mice show a reduction in calcification after treatment with the recombinant OPG–Fc protein, a synthesized version of OPG; however, there was no effect on atherosclerotic lesion size (Morony et al., 2008). OPG has been identified as a potential marker of atherosclerosis, as plasma levels of OPG increase with increasing severity of atherosclerosis (Hosbond et al., 2012, Kim et al., 2013). However, OPG may also be a marker of disease onset as LDLR^−/−^ mice have increased OPG levels upon disease onset, which did not increase with disease progression (Morony et al., 2008). This may not be the case in atherosclerosis patients; however, it is clear from the currently available literature that further studies are required if we are to fully elucidate the role of OPG in atherosclerosis and cardiovascular disease.

## 7 Osteoprotegerin in pulmonary arterial hypertension

More recently, OPG has been implicated as a potential mediator in the pathogenesis of PAH (Lawrie et al., 2008). Pulmonary arterial hypertension (PAH) is a fatal lung disease characterised by progressive pulmonary vascular remodelling, a key component of which is the proliferation and migration of pulmonary arterial smooth muscle cells (PASMCs). PAH is defined as pre-capillary pulmonary hypertension and is diagnosed by right heart catheterisation as an mean pulmonary artery pressure of greater than or equal to 25 mm Hg, a pulmonary arterial wedge pressure of less than 15 mm Hg, and a pulmonary vascular resistance (PVR) of greater than 240 dynes.s.cm^− 5^ (3 Woods Units (mm Hg/l.min)) (Hoeper et al., 2013). Pathologically, PAH is characterised by sustained vasoconstriction and the obliteration of the small pulmonary arteries and arterioles through medial hypertrophy, muscularisation, intimal fibrosis, and plexiform lesion formation (Tuder et al., 2013). Although the exact cause of PAH is currently unknown, endothelial dysfunction and pulmonary artery endothelial cell (PA-EC) apoptosis is thought to be an important early insult in disease pathogenesis. Subsequent pulmonary arterial smooth muscle cell (PASMC), PA-EC, and fibroblast proliferation and migration within the media of the pulmonary artery are then thought to drive the pulmonary vascular remodelling process (Tuder et al., 2013).

**Fig. 2:**
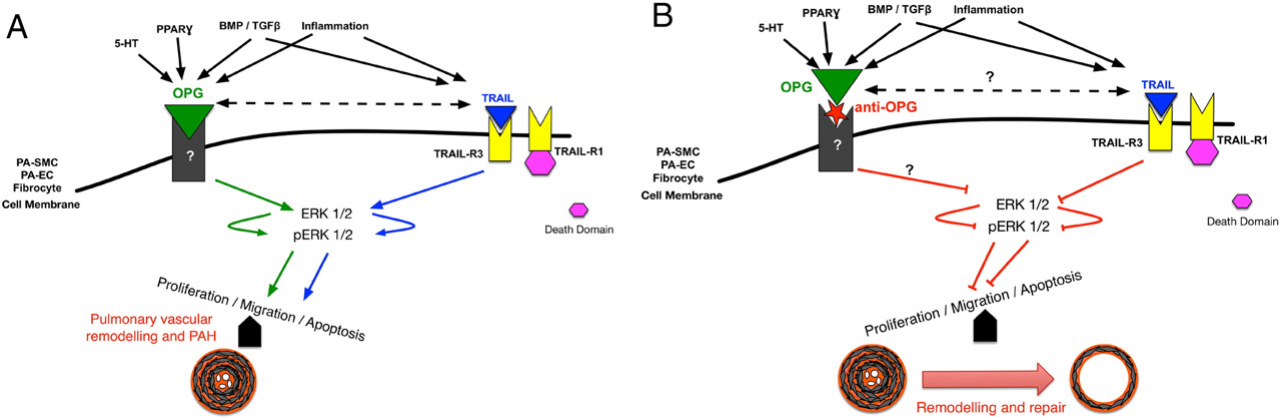
*Proposed model for OPG signalling events driving PAH pathogenesis.* (A) Multiple stimuli including 5-HT, inflammation and reduced BMPR2 stimulate the expression and release of OPG causing an increase in intracellular kinase signalling leading to the activation of multiple genes associated with PAH. This induces a pro-survival, migratory, and proliferative phenotype resulting promoting pulmonary vascular remodelling and PAH. (B) Inhibition of OPG reduces kinase expression and normalises the expression of the altered PAH-associated gene expression to reduce the anti-apoptotic, pro-proliferative phenotype and induce reverse pulmonary vascular remodelling to normalise pulmonary vascular resistance and PAH. The effect of OPG on TRAIL expression is unknown but unpublished research suggests additional links other than direct protein–protein interaction.

OPG is regulated by BMP signalling, serotonin, and interleukins, predominantly IL-1, which are important pathways implicated in the pathogenesis of PAH (Lawrie et al., 2008) (Fig. 2A). Immunohistochemical analysis of human lungs from patients with idiopathic PAH (IPAH) demonstrates increased OPG expression within pulmonary vascular lesions, and patients with IPAH also have increased serum levels of OPG, which correlate with prognostic markers and survival (Condliffe et al., 2012). PASMC isolated from patients with heritable PAH (hPAH) known to be harbouring BMPR2 mutations have increased expression of OPG, and the OPG-binding partners, TRAIL and RANKL, compared to cells from controls (Lawrie et al., 2008, Hameed et al., 2012). Normal, human PA-SMCs transfected with BMPR2 short interfering RNA (siRNA, to mimic the heterozygous loss of function BMPR2 mutation) demonstrated increased OPG release. Normal PASMC treated with 5HT and IL-1 also demonstrated a dose-dependent increase in OPG release (Lawrie et al., 2008). Functional studies have demonstrated that recombinant OPG induces both the proliferation and migration of PASMCs *in vitro*, in a dose-dependent manner suggesting that OPG may be a key downstream mediatory of disease pathogenesis (Fig. 2A).

## 8 Therapeutic potential of blocking osteoprotegerin for the treatment of pulmonary arterial hypertension

PAH describes a group of rapidly progressive conditions that share a common diagnosis of increased blood pressure in the lungs that over time the increased pressure causes heart failure. Pathologically the disease is driven by progressive pulmonary vascular remodelling that comprises a combination of sustained vasoconstriction and the obliteration of small pulmonary arteries through a process of cellular proliferation, intimal fibrosis, and the formation of angio-proliferative (plexiform) lesions (Tuder et al., 2013). Current treatments target vasoconstriction via the prostacyclin, endothelin, or nitric oxide pathways (Humbert et al., 2014). These drugs do little to address the underlying proliferative vascular disease and there remains no curative pharmacological treatment for PAH, with the 3 and 5 year survival for PAH in its idiopathic reported to be as low at 38% and 17%, respectively (Hurdman et al., 2012). We have described above a potential key role that OPG, which is found to be up-regulated within remodelled pulmonary arteries from patients with PAH, is regulated by multiple pathways important in PAH pathogenesis, and stimulates PASMC proliferation and migration and plays in the pathogenesis of PAH. Given this proposed active role in disease, we hypothesise that OPG is therefore a novel drug target. Since OPG is a naturally secreted protein and is already known to interact with several other proteins, we propose the best approach for targeting OPG to be a monoclonal anti-OPG antibody (Fig. 2B). This is an avenue that we are currently exploring with further pre-clinical studies currently underway.

## 9 Limitations of osteoprotegerin as a therapeutic target

Despite this promise, as with most therapeutic approaches, there are risks associated with the inhibition of OPG. Theoretically, blockade or inhibition of OPG may increase the availability of both RANKL and TRAIL. The most obvious risk associated with this being osteoporosis, or increased fracture risk due to the excess of RANKL–RANK-mediated bone reabsorption. Indeed the use of a recombinant form of OPG has previously been investigated as a therapeutic strategy to inhibit RANKL demonstrated promising early-phase promise in clinical trials (Bekker et al., 2001, Body et al., 2003); however, the emergence of denosumab (Bekker et al., 2001), a human anti-RANKL monoclonal antibody, superseded the development of recombinant OPG. However, these detrimental effects on bone can likely be tolerated in the context of PAH where life expectancy is substantially shortened and may be treated with other therapeutic approached. Furthermore, better understanding of the mechanism of action for OPG in PAH may identify a therapeutic approach to inhibit or block OPG while minimizing the impact on bone biology.

## Conflict of Interest Statement

AL has been funded through personal Fellowship awards from the Medical Research Council UK, Career Development Award (G0800318), and a British Heart Foundation, Senior Basic Science Research Fellow (FS/13/48/30,453). AL has been granted a UK patent GB2510524 (other territories pending) around the area of targeting OPG for the treatment of PAH and is a founding director of PH Therapeutics Ltd., a University of Sheffield spin-out company.
